# The Z-Drugs Zolpidem, Zaleplon, and Eszopiclone Have Varying Actions on Human GABA_*A*_ Receptors Containing γ1, γ2, and γ3 Subunits

**DOI:** 10.3389/fnins.2020.599812

**Published:** 2020-11-19

**Authors:** Grant Richter, Vivian W. Y. Liao, Philip K. Ahring, Mary Chebib

**Affiliations:** Brain and Mind Centre, Sydney Pharmacy School, The University of Sydney, Sydney, NSW, Australia

**Keywords:** GABA_*A*_ receptors, Z-drugs, modulators, γ1 subunit, γ3 subunit, zolpidem, zaleplon, eszopiclone

## Abstract

γ-Aminobutyric-acid type A (GABA_*A*_) receptors expressing the γ1 or γ3 subunit are only found within a few regions of the brain, some of which are involved in sleep. No known compounds have been reported to selectively target γ1- or γ3-containing GABA_*A*_ receptors. Pharmacological assessments of this are conflicting, possibly due to differences in experimental models, conditions, and exact protocols when reporting efficacies and potencies. In this study, we evaluated the modulatory properties of five non-benzodiazepine Z-drugs (zaleplon, indiplon, eszopiclone, zolpidem, and alpidem) used in sleep management and the benzodiazepine, diazepam on human α1β2γ receptors using all three γ subtypes. This was accomplished using concatenated GABA_*A*_ pentamers expressed in *Xenopus laevis* oocytes and measured via two-electrode voltage clamp. This approach removes the potential for single subunits to form erroneous receptors that could contribute to the pharmacological assessment of these compounds. No compound tested had significant effects on γ1-containing receptors below 10 μM. Interestingly, zaleplon and indiplon were found to modulate γ3-containing receptors equally as efficacious as γ2-containing receptors. Furthermore, zaleplon had a higher potency for γ3- than for γ2-containing receptors, indicating certain therapeutic effects could occur via these γ3-containing receptors. Eszopiclone modulated γ3-containing receptors with reduced efficacy but no reduction in potency. These data demonstrate that the imidazopyridines zaleplon and indiplon are well suited to further investigate potential γ3 effects on sleep *in vivo.*

## Introduction

γ-Aminobutyric-acid type A (GABA_*A*_) receptors are ligand-gated ion channels that mediate most inhibitory responses in the brain. These receptors are made up of five building block subunits, and in mammals, there are nineteen identified subunits, α1-6, β1-3, γ1-3, δ, ε, θ, π, and ρ1-3 (Sieghart and Savic, 2018). Receptors typically form from two α, two β, and one of either γ or δ with the most widely expressed combination made from two α1, two β2/3, and one γ2, denoted as α1β2/3γ2 (Olsen and Sieghart, 2008). Distinctive GABA_*A*_ receptor subtypes are found based on their cellular and anatomical locations and behave differently in response to agonists and modulating compounds.

**FIGURE 1:**
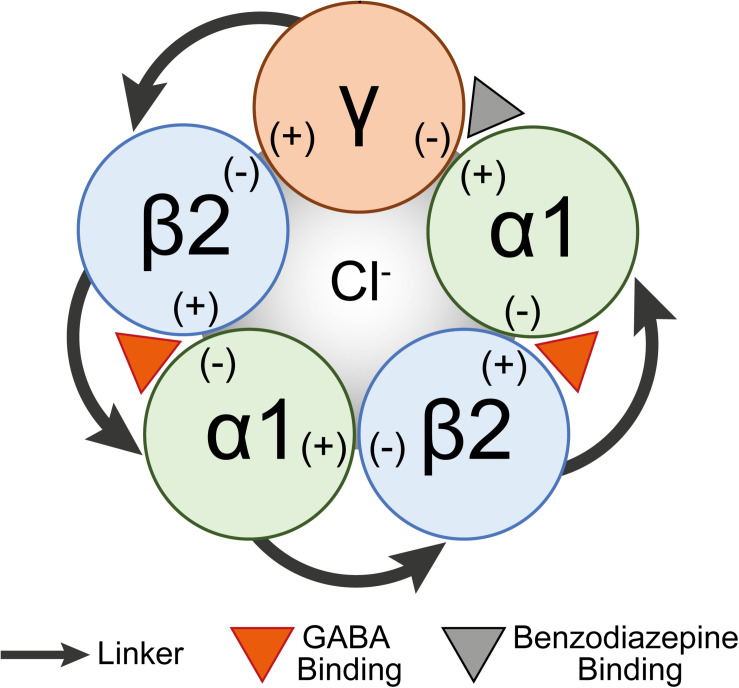
A schematic diagram of a concatenated pentamer GABA_*A*_ receptor construct. Linkers concatenating subunits are shown as arrows. The GABA binding site (orange arrowhead) is shown between the β2(+) and α(–) interfaces and the benzodiazepine binding site (gray arrowhead) is between the α1(+) and γ(–) interfaces.

Each GABA_*A*_ subunit contains a principal side (+) and a complimentary side (−). GABA binding within the β(+) and α(−) interface induces a conformational change in the receptor channel allowing Cl^–^ ions to pass into the cell to hyperpolarize neurons and make action potentials less likely (Figure 1). Benzodiazepines and Z-drugs allosterically modulate GABA_*A*_ receptors making the frequency of Cl^–^ channel opening more likely. These drugs bind to the interface within the α(+) and γ(−) (Sigel and Buhr, 1997; Zhu et al., 2018) to reduce the brain’s excitability and thus are primarily prescribed for their effects as anxiolytics, hypnotics, anti-epileptics, and muscle relaxants. Z-drugs are the most commonly prescribed treatment for insomnia and compared with benzodiazepines they more closely induce normal physiological sleep (Klimm et al., 1987; Fleming et al., 1988). However, it is still not precisely characterized which regions Z-drugs act on to induce sleep.

While three γ subunits exist, the actions of benzodiazepines and Z-drugs have typically been associated with the γ2 subunit with little information available for γ1 and γ3 subunits. The γ1 or γ3-subunits are found in at most 10 or 15% of GABA_*A*_ receptors, respectively (Quirk et al., 1994; Benke et al., 1996; Sieghart and Sperk, 2002). Temporally, the γ2 subunit is expressed throughout all stages of development, while γ1 subunit expression peaks around birth and γ3 subunit expression peaks in 2-week old animals (Laurie et al., 1992; Allen Institute for Brain Science, 2008) GABA_*A*_ receptors with a γ1 subunit have been detected mainly in the amygdala, basal ganglia, hypothalamus, thalamus, and in astrocytes, while receptors with γ3 subunits show some expression in the basal ganglia, thalamus, and midbrain (Bovolin et al., 1992; Quirk et al., 1994; Pirker et al., 2000; Sieghart and Sperk, 2002; Hertz and Chen, 2010). The thalamus and hypothalamus regions are intricately involved in the maintenance of the sleep-wake cycle (Gent et al., 2018) and Z-drugs have been shown to affect clusters of nuclei in these regions (Jia et al., 2009; Kumar et al., 2011; Uygun et al., 2016). Hence, it is a genuine possibility that γ1- or γ3-containing receptors could also play a role in the hypnotic effects of Z-drugs. Indeed the interface between α(+)/γ(−) is believed to be sensitive to benzodiazepine binding in both γ1 and γ3 containing receptors, though some ligands might have lower potencies and/or efficacies because of amino acid sequence differences (Knoflach et al., 1991; Sieghart, 1995; Khom et al., 2006).

Although some Z-drugs have been evaluated on γ1 or γ3-containing GABA_*A*_ receptors, it is difficult to conclude any clear effects mediated from these subunits as there is conflicting literary evidence of the modulative ability of Z-drugs. This may be due to differences in experimental models, conditions, and exact protocols reported for efficacy and potencies of these compounds. Furthermore, studies that utilize *Xenopus laevis* oocytes to investigate the pharmacology of Z-drugs have conflicting results potentially due to using single subunit cRNAs to express recombinant receptors. Using single subunit cRNAs in a heterologous expression system can potentially result in a mix of receptor populations. For example, if unlinked cRNAs for α1, β2, and γ subunits are injected into a cell, there is potential for GABA_*A*_ receptors to assemble from only α1 and β2 with two different stoichiometries [i.e., (α1)_2_(β2)_3_ or (α1)_3_(β2)_2_], potentially confounding results. Therefore, our group has recently optimized receptor concatenation technology to ensure a single receptor subtype population with assembly in the correct orientation (Liao et al., 2019).

**FIGURE 2:**
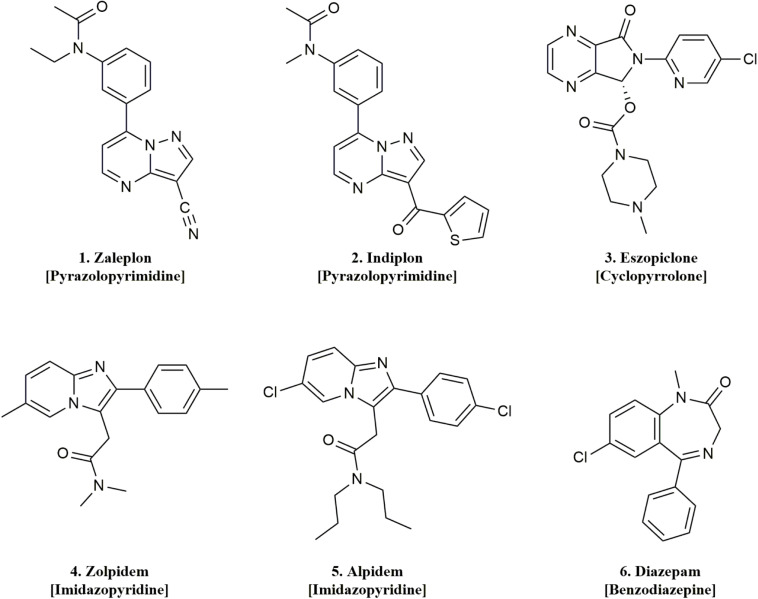
Chemical structures and classes of the drugs used in this study.

In the present study, we systematically evaluated the pharmacology of five Z-drugs including the pyrazolopyrimidines (zaleplon and indiplon), cyclopyrrolones (zopiclone and its isolated *S*-enantiomer eszopiclone), and imidazopyridines (zolpidem and alpidem), along with diazepam on γ1, γ2, and γ3 concatenated pentameric GABA_*A*_ receptors (Figure 2). We found that zaleplon, indiplon, and eszopiclone show comparable efficacy and potency on γ3 as γ2-containing receptors. Furthermore, zolpidem and alpidem modulate γ2 receptors with exclusive selectivity at concentrations below 10 μM. These data clarify conflicting observations and provide further insight into the receptor subtype populations targeted by Z-drugs, and identifies zaleplon, indiplon, and possibly eszopiclone as useful tools for further studies that understand the role γ3-containing receptors in sleep.

## Materials and Methods

### Materials

GABA, diazepam, alpidem, and all salts and chemicals not specifically mentioned were purchased from Sigma-Aldrich. Zolpidem was purchased from Chemieliva (Yubei District, Chongqing, China), zaleplon was purchased from Alomone Labs (Jerusalem, Israel), eszopiclone was purchased from Clearsynth (NJ, United States), and indiplon was purchased from Tocris (VIC, Australia). Human cDNA for α1 β2, γ1,2,3 GABA_*A*_ receptor subunits were gifts from Saniona A/S. Oligonucleotides were purchased from Sigma-Aldrich. Restriction enzymes, Q5 polymerase, T4 DNA ligase, and 10-beta competent *Escherichia coli* were from New England Biolabs (Ipswich, MA, United States). Collagenase A was purchased from Roche (Basel, Switzerland). DNA purification kits were from Qiagen (Hilden, Germany). The QuickChange II Site-Directed Mutagenesis Kit was from Agilent Technologies (Santa Clara, CA, United States). The mMessage mMachine T7 transcription kit–were purchased from Thermo Fisher Scientific (Waltham, MA, United States).

### Molecular Biology

To ensure homogenous receptor populations and subunit orientation, we used concatenated receptors expressed in *X. laevis* oocytes. Concatenated pentameric constructs were created using the subunits γx-β2-α1-β2-α1 (where *x* = 1, 2, or 3). A detailed description of the creation of concatenated receptor constructs has been previously described (Liao et al., 2019). Briefly, natural restriction sites *Bam*HI, *Hin*dIII, and *Kpn*I restriction sites in the γ1, 2, 3, β2, or α1 subunits were removed through silent mutations using site-directed mutagenesis. Linker sequences of 13 amino acids inserted between the natural C-terminal in the transmembrane segment 4 of the γ subunit and the N-terminal leucine anchor of the β subunit through standard PCR reactions and subunit cDNA ligated together (corresponding to a total linker length of 28 total amino acids between subunits) This was found to be an optimal length for relatively pure receptor expression and orientation without compromising function (Liao et al., 2019). Inserted linker lengths are as follows γx-13a-β2-27a-α1-18a-β2-27a-α1. *E. coli* bacteria were hosts for plasmid amplification and plasmid purification was performed using standard kits. RNA was produced from DNA using the mMessage mMachine T7 Transcription kit (Thermo Fisher Scientific, Waltham, MA, United States), but due to the size of the pentameric constructs (>10 kb), guanosine triphosphate concentration was increased to give a final cap analog to guanosine triphosphate ratio of 2:1.

### Expression of GABA_*A*_ Receptors in *X. laevis* Oocytes

The collection and preparation of oocytes were done as previously described (Ahring et al., 2016). Briefly, ovarian lobes were removed from anesthetized adult *X. laevis* following protocol approval by the Animal Ethics Committee of The University of Sydney (AEC No. 2016/970) in accordance with the National Health and Medical Research Council of Australia code for the care and use of animals. Oocytes were prepared by slicing lobes into small pieces and defolliculated through collagenase A treatment. Stage V and VI oocytes were injected with around 50 nL of 0.5 ng/nL RNA for each concatenated construct or α1/β2 subunits in a 1:1 ratio and incubated for 3–4 days at 18°C in modified Barth’s solution (96 mM NaCl, 2.0 mM KCl, 1 mM MgCl_2_, 1.8 mM CaCl_2_, 5 mM HEPES, 2.5 mM sodium pyruvate, 0.5 mM theophylline, and 100 μg/mL gentamicin; pH 7.4).

### Electrophysiological Recordings Using Two-Electrode Voltage Clamp

This technique was performed as previously described (Ahring et al., 2016, 2018; Liao et al., 2019). Briefly, oocytes sit in a custom-built chamber and continuously perfused with a saline solution, ‘ND96’ (96 mM NaCl, 2 mM KCl, 1 mM MgCl_2_, 1.8 mM CaCl_2_, and 10 mM HEPES; pH 7.4). Glass electrode pipettes were filled with 3 M KCl, with resistances ranging from 0.4 to 2 MΩ. Oocytes were clamped to −60 mV using an Axon GeneClamp 500B amplifier (Molecular Devices). Currents were filtered at 20 Hz with a four-pole low pass Bessel filter (Axon GeneClamp 500 B) and digitized by a Digidata 1440A (Molecular Devices). Sampling was taken at 200 Hz and analyzed using pClamp 10.2 suite (Molecular Devices).

Stock solutions of 3.16 M GABA in ultrapure water and drug solutions of 100 mM in DMSO were stored at −20°C and aliquoted to avoid repeated freeze-thaw cycles. Each recording day, a fresh stock was used to prepare dilutions. The maximal concentration of DMSO in final drug ND96 solutions was <0.1%.

### Experimental Design

GABA concentration-response curves were determined for each construct as follows. To ensure RNA expression and reproducibility, a set of control applications were first applied consisting of three applications of 40 μM GABA, one 316 μM application, and three more 40 μM applications. After this, ten solutions of GABA each increasing in concentration by a factor of 3.16 were used starting with 100 nM and ending with 3.16 mM. Applications lasted for 30 s and were followed by 2–5 min of washout. EC_50_ and EC_10_ were calculated from this curve.

The drug modulation experiments were done as follows. Like the GABA dose-response curves, first, a set of three control applications were run consisting of GABA EC_10_, then a maximal response of GABA 3.16 mM, followed by three more GABA EC_10_. Before the application of modulators, EC_10_ was confirmed by comparing the ratio of the current of the last control application to the maximal response current. For each drug, 6 concentrations increasing by a factor of 10, ranging from 0.1 nM to 10 μM, were co-applied with GABA EC_10_ for 30 s followed by 2–5 min of washout.

### Data and Statistical Analysis

The final dataset was from a minimum of four experiments and a minimum of two different *X. laevis* donors. Raw traces were analyzed using pClamp 10.2. Episodic traces for each application were overlaid and the baseline was subtracted. Peak current amplitude was quantified by measuring maximum inward current for each response. Peak current amplitudes (I) were fitted to the Hill equation and normalized to the maximal fitted response (Imax). The calculated E_*max*_ response is expressed as a percentage of the current obtained through GABA EC_10_ (actual GABA control percentage for each experiment is listed in Table 1). The E_*max*_ response and EC_50_ values were calculated by using non-linear regression to fit the data to the Hill equation in a monophasic model with three variables (top, bottom, EC_50_) using GraphPad Prism 8. Efficacy at infinitely low compound concentration was set to 0, and the slope was constrained to 1. For GABA concentration-response curves, the slope was unconstrained and listed in Table 1. Means are reported ± one SD. To compare differences in E_*max*_ response, EC_50_ values within drug groups and across γ3 and γ2 receptors, or to compare γ1/3 receptor responses at 10 μM with binary α1β2 receptors, one-way ANOVAs were run with Sidak multiple comparisons test. F tests, respectively, are [*F* (7, 48) = 32.77, *p* < 0.0001] and [*F* (7, 48) = 75.69, *p* < 0.0001], and [*F* (14,57) = 48.68, *p* < 0.0001]. All reported statistically significant comparisons within the results section are *p* < 0.01.

**TABLE 1:** Potency and efficacy of Z-drugs on GABA_*A*_ receptors with varying γ subunits.

**GABA_*A*_ Construct**		**Zaleplon**	**Indiplon**	**Zolpidem**	**Alpidem**	**Eszopiclone**	**Diazepam**
γ1 (α1β2γ1)	Mean GABA_*Control*_ (%)	10.7 ± 4	9.6 ± 4	10.6 ± 4	10.8 ± 4	12.5 ± 4	12.2 ± 4
	E_*max*_ (%)	No fit	No fit	No fit	No fit	No fit	No fit
	Modulation at 10 μM	126 ± 3	22 ± 7	15 ± 6	44 ± 10	18 ± 19	43 ± 6
	EC_50_	No fit	No fit	No fit	No fit	No fit	No fit
	Log(EC_50_)	No fit	No fit	No fit	No fit	No fit	No fit
	*n* value	6	4	5	5	5	5
γ2 (α1β2γ2)	Mean GABA_*Control*_ (%)	8.2 ± 3	8.5 ± 2	9.5 ± 2	10.4 ± 3	8.6 ± 3	9.3 ± 3
	E_*max*_ (%)	307 ± 34	231 ± 18	487 ± 66	512 ± 80	356 ± 37^†^	284 ± 23^†^
	Modulation at 10 μM	305 ± 59	238 ± 31	484 ± 124	542 ± 176	349 ± 70	272 ± 46
	EC_50_	203 nM*	13.1 nM^∗^	230 nM	502 nM	301 nM	139 nM*
	Log(EC_50_)	−6.69 ± 0.2*	−7.88 ± 0.2*	−6.64 ± 0.3	−6.3 ± 0.4	−6.52 ± 0.2	−6.86 ± 0.2*
	*n* value	6	8	5	7	6	5
γ3 (α1β2γ3)	Mean GABA_*Control*_ (%)	10.7 ± 2	12 ± 1	10.2 ± 4	10.3 ± 1	10 ± 2	10.4 ± 2
	E_*max*_ (%)	277 ± 26	263 ± 18	No fit	No fit	207 ± 20^†^	168 ± 24^†^
	Modulation at 10 μM	262 ± 53	249 ± 14	101 ± 37	126 ± 16	199 ± 26	156 ± 35
	EC_50_	56 nM*	47.8 nM*	No fit	No fit	554 nM	1920 nM*
	Log(EC_50_)	−7.25 ± 0.2*	−7.32 ± 0.2*	No fit	No fit	−6.26 ± 0.2	−5.72 ± 0.3*
	*n* value	8	7	5	8	8	8

*Mean values shown ± SD, no fit indicates data could not fit to hill equation. ^†^*Post hoc* test *p* ≤ 0.0001 comparing E_*max*_ (%) within drug groups for γ2 vs. γ3 receptors {one-way ANOVA [*F* (7,48) = 32.77, *p* < 0.0001]}. ^∗^*Post hoc* test *p* ≤ 0.0001 comparing Log(EC_50_) within drug groups for γ2 vs. γ3 receptors {one-way ANOVA [*F* (7,48) = 75.69, *p* < 0.0001]}.*

## Results

### GABA Response of Concatenated γ1-, γ2-, and γ3-Containing GABA_*A*_ Receptors

To ensure homogenous receptor populations and subunit orientation, we used concatenated receptors expressed in *X. laevis* oocytes. Concatenated pentameric constructs were created using the subunits γ-β2-α1-β2-α1 (where γ = γ1, γ2, or γ3). Subunits were linked with artificial linker sequences optimized to give relatively pure receptor expression and orientation without compromising function (Liao et al., 2019).

**FIGURE 3:**
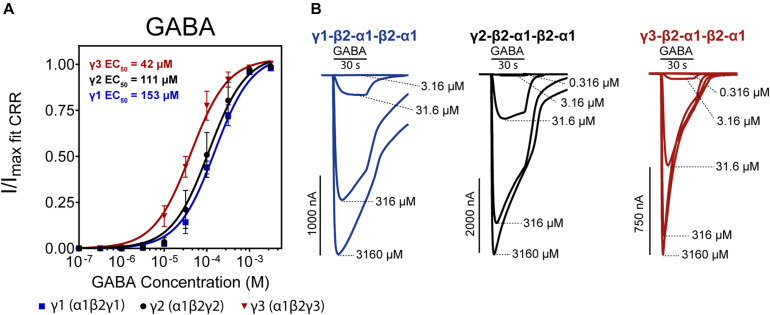
**(A)** Normalized GABA concentration-response curves of α1β2γx receptors (*x* = 1,2,3) expressed as concatenated pentamers in *Xenopus laevis* oocytes measured via two-electrode voltage clamp. Oocytes were injected with 50 nL of 0.5 ng/nL cRNA for each concatenated construct. Datapoints are depicted as means ± SD (*n* = 12–14). Data were fitted by non-linear regression to the Hill equation with an unconstrained Hill slope. Log(EC_50_) and Hill slope parameters are as follows; γ1 Log(EC_50_) = –3.82 ± 0.07 Hill slope = 1.22 ± 0.32, γ2 Log(EC_50_) = –3.96 ± 0.10 Hill slope = 1.19 ± 0.55, γ3 Log(EC_50_) = –4.38 ± 0.07 Hill slope = 1.36 ± 0.63. **(B)** Representative traces of each construct with indicated concatenated subunit combination. Application bars designate 30 s application time and concentrations of GABA are indicated at the peak of each trace.

We first measured the concentration-response for GABA on each construct (Figure 3A). Upon visual inspection of representative traces (Figure 3B), γ1 and γ2 receptors presented similar current decay profiles at the highest GABA concentrations while the γ3 receptor showed a shorter current decay time. This could indicate that γ3 receptors undergo a higher degree of desensitization upon prolonged GABA exposure than the γ1 and γ2 receptor counterparts.

The three receptor subtypes presented EC_50_ values in the range of 42–153 μM with γ3 being the most sensitive and γ1 being the least sensitive to GABA. The value for the γ2-containing concatenated receptor (EC_50_ of 111 μM) is in good agreement with Liao et al. (2019). Previously reported GABA EC_50_ values using single subunit injections of GABA_*A*_ γ1, 2, 3 cRNAs in *X. laevis* oocytes show substantial variations in obtained GABA potencies ranging from 5–100 μM, but generally, γ3 receptors appear more sensitive to GABA (Knoflach et al., 1991; Wafford et al., 1993; Ebert et al., 1994; Khom et al., 2006; Esmaeili et al., 2009).

### Comparing the Efficacy of Modulators Between GABA_*A*_ Receptor Subtypes

Positive allosteric modulators work by increasing the open-state probability of a receptor in the presence of an endogenous ligand (GABA). If the receptor is already at its maximal open-state probability, then the modulator will have no additional effect. For all γ-containing GABA_*A*_ receptors, applications of high concentrations of GABA (>1 mM) are typically able to reach activation levels close to the maximal open-state probability, hence, modulators show no efficacy under conditions with high GABA concentrations. Whereas allosteric modulators, by definition, should not gate the receptor in the absence of GABA, substantial modulatory efficacies can be observed as GABA concentrations are lowered toward zero. Therefore, any efficacy of modulators described in percent will depend entirely on the selected concentration of the endogenous ligand. Low concentrations of GABA co-applied with modulators will yield large modulatory percent changes. Conversely, higher concentrations of GABA co-applied with modulators give small percent changes.

For our experiments, we selected to co-apply modulators with a GABA_*control*_ concentration that yields 10% of the maximum response (EC_10_) at the given receptor. Modulator efficacy is reported as a percent change of evoked current amplitude relative to the GABA_*control*_ application alone. To directly compare modulator efficacy across different receptors, it was critical that each experiment is run as close as possible to the EC_10_ of that receptor subtype. Due to GABA potency variations both between batches of oocytes and between individual oocytes, each experiment began with a full GABA concentration-response to determine EC_10_. Then for each oocyte, a set of 3 control applications at EC_10_ followed by a max GABA application, followed by three more EC_10_ applications were applied to confirm that the chosen GABA_*control*_ concentration yielded ∼10% of the maximum response. Any oocytes responding outside this narrow range (10% ± 5) were discarded before continuing with modulator experiments. GABA_*control*_ variation is reported in Table 1.

### Modulatory Potency and Efficacy of Z-Drugs and Diazepam on GABA_*A*_ γ1-, γ2-, and γ3-Containing Receptors

**FIGURE 4:**
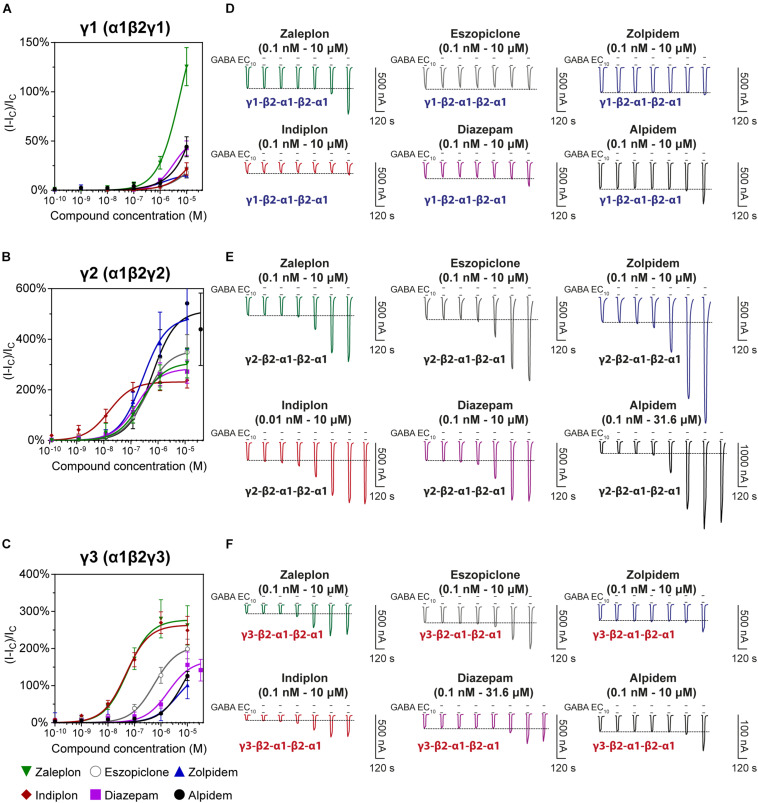
Modulatory actions of zaleplon, indiplon, eszopiclone, diazepam, zolpidem, and alpidem, on GABA evoked Cl^–^ currents measured in human **(A)** α1β2γ1, **(B)** α1β2γ2, and **(C)** α1β2γ3 GABA_*A*_ receptors expressed in *Xenopus laevis* oocytes measured via two-electrode voltage-clamp. The data are expressed as a percentage potentiation of GABA EC_10_ and are means ± SD (*n* = 4–8 from at least 2 separate *Xenopus laevis* donors). Data points were fitted to the Hill equation with bottom set to 0 and slope constrained to 1. **(D–F)** Representative traces illustrating modulator concentration-response experiments.

We examined the modulatory effects of the non-benzodiazepines ‘Z-drugs’ (zaleplon, indiplon, eszopiclone, zolpidem, and alpidem) and the benzodiazepine, diazepam on GABA_*A*_ receptors with varying γ subunits (Figures 4A–C). Representative traces for each compound and receptor subtype are shown in Figures 4D–F. Concentrations ranging from 10^–10^ to 10^–5^ M were co-applied with GABA EC_10_. Full experimental results with Log(EC_50_) ± SD are listed in Table 1. Unless stated otherwise, all reported statistically significant comparisons have a *p* < 0.01.

### Pyrazolopyrimidines

**FIGURE 5:**
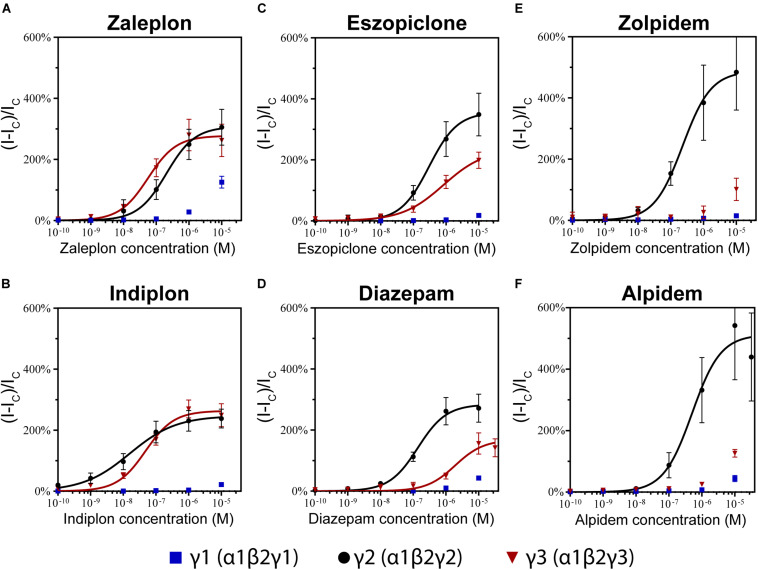
Modulatory actions of **(A)** zaleplon, **(B)** indiplon, **(C)** eszopiclone, **(D)** diazepam, **(E)** zolpidem, and **(F)** alpidem, on GABA evoked Cl^–^ currents measured in human α1β2γ1, α1β2γ2, and α1β2γ3 GABA_*A*_ receptors expressed in *Xenopus laevis* oocytes measured via two-electrode voltage-clamp. The data are expressed as a percentage potentiation of GABA EC_10_ and are means ± SD (*n* = 4–8 from at least 2 separate *Xenopus laevis* donors). Data points were fitted to the Hill equation with bottom set to 0 and slope constrained to 1.

The pyrazolopyrimidines, zaleplon and indiplon, showed a reverse potency preference for γ2 and γ3 receptors (Figures 5A,B). Zaleplon had a ∼4-fold greater potency at γ3 receptors compared with γ2 (EC_50_ of approximately 50 vs. 200 nM), while indiplon had a ∼4-fold greater potency for γ2 vs. γ3 (10 vs. 45 nM). Neither compound had statistically significant different efficacies when γ2 was replaced by γ3 with E_*max*_ both in the range of ∼250–300%. On γ1 receptors, neither compound showed sufficient potency to enable fitting to the Hill equation within the concentration range tested. At the highest concentration applied (10 μM), zaleplon elicited a modulatory response of 125% and indiplon, 20%.

In support of these findings, previous competitive binding studies using Ro15-4513 have suggested that zaleplon binds to γ3 GABA_*A*_ receptors with an eightfold higher affinity than when γ2 is present (Dämgen and Lüddens, 1999). However, efficacy and potency have only been studied for the α2β2γ3 receptor which shows 10-fold less potency than what we have seen on α1β2γ3 with an EC_50_ of ∼500 nM.

### Eszopiclone and Diazepam

The cyclopyrrolone, eszopiclone, and the benzodiazepine, diazepam modulated both γ2 and γ3 containing receptors with varying potency and efficacies and did not significantly modulate γ1 containing receptors (Figures 5C,D). Substituting the γ3 receptor for γ2 had no statistically significant difference on eszopiclone’s potency (in the range of 300–500 nM), but diazepam had a ∼15-fold reduction (1900 vs. 150 nM). Both compounds had ∼1.5-fold reductions in E_*max*_ when γ3 replaced γ2 (300 vs. 200%). At 10 μM, eszopiclone modulated γ1 receptors by 20% and diazepam by 40% above GABA EC_10_.

No literary data are available for eszopiclone, however, the racemic mixture zopiclone has been investigated. In a competitive binding study from Dämgen and Lüddens (1999), a marginal reduction in binding affinity was observed for zopiclone when γ3 was replaced by γ2. Yet, in another study zopiclone was observed to modulate α1β2γ3 receptors with comparable efficacy and potency to that of α1β2γ2 (Davies et al., 2000). Hence, eszopiclone and zopiclone seem to behave in a similar fashion at γ2- and γ3 containing receptors A previous study of diazepam on recombinant α1β2γ3 GABA_*A*_ receptors shows good agreement for the potency (EC_50_ of 1.95 μM), but they observed no reduction in E_*max*_ comparing α1β2γ2 vs. α1β2γ3 receptors (Lippa et al., 2005).

### Imidazopyridines

The imidazopyridines, zolpidem, and alpidem were selective for the γ2 subunit, not showing significant potencies to be able to estimate an EC_50_ from fitting to the Hill equation for γ1 and γ3 receptors (Figures 5E,F). Zolpidem and alpidem had E_*max*_ on γ2 receptors ranging from 475–550%. Zolpidem’s EC_50_ on γ2 receptors was 230 nM and alpidem’s 500 nM. On γ3 receptors, both compounds had a measured response at concentrations of 10 μM of near 125% of GABA EC_10_. Neither compound showed robust efficacy on γ1 containing receptors. At 10 μM, zolpidem elicited a response of 15% and alpidem 40% above GABA_*control*_. Overall this data indicates that zolpidem’s pharmacological activity is likely to be related only to the γ2 subunit.

Zolpidem’s selectivity for the γ2 subunit below 10 μM correlates with previous studies both on the binding for the γ1 (Benke et al., 1996) and γ3 subunit (Herb et al., 1992; Lüddens et al., 1994; Tögel et al., 1994; Hadingham et al., 1995; Sieghart, 1995; Dämgen and Lüddens, 1999), and with measurements in oocytes showing 20% or less efficacy (Wafford et al., 1993; Mckernan et al., 1995; Khom et al., 2006). These observations contrast with studies using HEK293 cells expressing α1βγ1 receptors observing zolpidem potentiating near 50–75% (Puia et al., 1991) and with an EC_50_ around 200 nM (Esmaeili et al., 2009).

### α1β2 Binary Receptors

**FIGURE 6:**
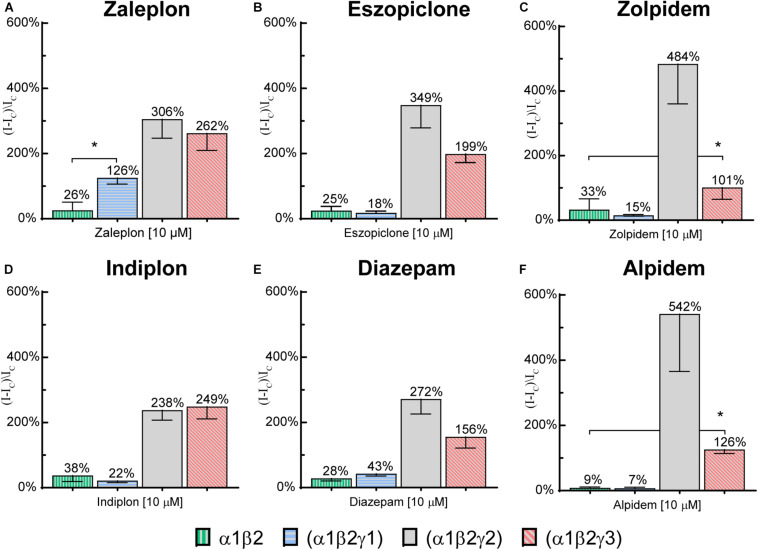
Modulatory actions of 10 μM **(A)** zaleplon, **(B)** indiplon, **(C)** eszopiclone, **(D)** diazepam, **(E)** zolpidem, and **(F)** alpidem, on GABA evoked Cl^–^ currents measured in human α1β2γ1, α1β2γ2, α1β2γ3, and binary α1β2 GABA_*A*_ receptors expressed in *Xenopus laevis* oocytes measured via two-electrode voltage-clamp. Data are expressed as percentage potentiation of GABA EC_10_ and are means ± SD (*n* = 3–8 from at least 2 separate *Xenopus laevis* donors). One-way ANOVA with *post hoc* Sidak multiple comparisons test were calculated between all compounds on γ1 vs. α1β2 and between zolpidem and alpidem on γ3 vs. α1β2; *p < 0.01.

To investigate whether the modulation observed at high compound concentrations on γ1 or γ3 receptors was specific to the γ subunit, 10 μM of each compound was applied to α1β2 binary receptors. Potentiation values are depicted along with the respective values at the γ-containing receptors in Figure 6. All 6 tested compounds elicited small responses on α1β2 receptors, with mean values ranging from 10–35%. Zaleplon was the only compound to show significantly higher γ1 receptor modulation above the value seen for α1/β2 receptors (*p* < 0.01) indicating that potentiation observed is specific to the γ1 subunit. Importantly, all tested compounds showed significantly higher potentiation values at γ3 receptors compared with α1β2 receptors (*p* < 0.01) indicating that modulation is specific to γ3.

## Discussion

In this study, we examined the effectiveness of the Z-drugs (zaleplon, indiplon, eszopiclone, zolpidem, and alpidem) and the benzodiazepine, diazepam on GABA_*A*_ receptors containing γ1, γ2, or γ3 subunits under highly controlled experimental conditions. We used concatenated pentamers expressed in *Xenopus laevis* oocytes to reduce the potential of confounding mixed receptor populations arising when single subunits are injected (Boileau et al., 2002; Sigel et al., 2006; Ahring et al., 2016; Liao et al., 2019). Furthermore, all experiments were performed identically for each oocyte. Modulators were co-applied with a GABA_*control*_ concentration eliciting 10% of the maximum response.

### α1β2γ2 Receptors

All the tested drugs are efficient and potent modulators of γ2 receptors. Maximum efficacies ranged from 250–500% with the most and least efficacious being alpidem and indiplon, respectively. Potencies ranged from 10–500 nM with the most and least potent being indiplon and alpidem, respectively. In general, our results are within the range of variation from previous studies (Puia et al., 1991; Davies et al., 2000; Sanna et al., 2002; Petroski et al., 2006).

### α1β2γ1 Receptors

None of the Z-drugs exhibited sufficient potency within the tested concentration range to allow reliable fitting of the data to the Hill equation at γ1 receptors. At the highest tested concentration (10 μM) zaleplon had an efficacy of 125%. This contrasts the structurally similar compound, indiplon which at the same concentration did not affect α1β2γ1 receptors. Notably, zaleplon’s modulation was likely specific to the γ1 subunit, as the same concentration applied to α1/β2 receptors only elicited 25% above GABA_*control*_. It remains a possibility that even higher concentrations of zaleplon could reveal further robust modulation at γ1-containing receptors. However, we generally chose to limit the concentration range tested to a maximum of 10 μM to avoid issues with compound solubility and potential interfering efficacies from binding to secondary modulatory sites as previously described for diazepam (Walters et al., 2000; Sieghart, 2015; Masiulis et al., 2019).

There is some discrepancy regarding γ1-containing receptors and zolpidem in the literature. Several studies observed that zolpidem displays no binding (Benke et al., 1996), or low maximum efficacies below 20% in α1βγ1 (Khom et al., 2006) and α2βγ1 receptors (Wafford et al., 1993; Mckernan et al., 1995) expressed in *X. laevis* oocytes. In contrast, other studies using HEK293 cells expressing α1βγ1 receptors observe zolpidem potentiations of near 50–75% (Puia et al., 1991) with an EC_50_ around 200 nM (Esmaeili et al., 2009). Differences in the experimental protocol, expression systems, assembling receptor populations, or chosen GABA control concentration may account for some of these divergences. Nevertheless, our data showing that zolpidem and the structurally similar compound, alpidem have negligible effects on concatenated pentameric α1β2γ1 receptors align with the findings that these compounds do not modulate γ1-containing receptors.

While definitive high-resolution crystal or Cryo-EM structures of GABA_*A*_ receptors with bound diazepam exist (Zhu et al., 2018), they are still lacking for Z-drugs. Mutational studies and molecular modeling have provided insights into the nature of the important amino acids determining Z-drugs’ binding within the α1(+)–γ2(−) interface. The necessary His101 residue on the α1(+) interface is a well-characterized component of the benzodiazepine binding site (Wieland et al., 1992; Benson et al., 1998; Mckernan et al., 2000), but there are also important residues on the γ2(−) side. The amino acids Met130 and Phe77 have interactions with zolpidem, and mutating one or more of these abolishes binding (Buhr and Sigel, 1997; Wingrove et al., 1997). These residues are not present on the γ1 subunit, yet introducing them into the γ1 subunit does not fully restore zolpidem binding (Wingrove et al., 1997). Furthermore, the γ2 Phe77 mutation when expressed in the mouse eliminated zolpidem (but not flurazepam) dependent sedation and decreases motor exploration (Cope et al., 2004). Overall, our data pose the question of whether any of the tested drugs bind efficiently to α1-γ1 interfaces within the tested concentration range.

### α1β2γ3 Receptors

Zaleplon, indiplon, and eszopiclone modulate γ3-containing GABA_*A*_ receptors at therapeutically relevant doses while diazepam, zolpidem, and alpidem do not. On α1β2γ3 receptors, zaleplon has equal efficacy compared with α1β2γ2, and a four-fold increase in potency. The structurally similar indiplon was also equally as efficacious on γ3- as γ2 containing receptors, but with reduced potency indicating that small differences in pyrazolopyrimidines can alter selectivity preferences between γ3- and γ2-containing GABA_*A*_ subunits. Eszopiclone potentiates α1β2γ3 receptors with equal potency to α1β2γ2, but with a 1.5-fold reduction in efficacy. Overall these data indicate that even though classes of Z-drugs are quite similar, the arylamide moiety located at C4 of the pyrazolopyrimidines may be important for binding to the γ3 subunit.

Interestingly, high concentrations of zolpidem and alpidem potentiated GABA at γ3 receptors. This effect is specific to the γ3 subunit, as the same concentration applied to α1/β2 receptors elicited little response. Previous competitive binding studies using high-affinity benzodiazepine site ligands such as flunitrazepam or Ro-154513 have indicated that zolpidem has no binding to the classical γ3 receptor benzodiazepine site (Herb et al., 1992; Lüddens et al., 1994; Tögel et al., 1994; Hadingham et al., 1995; Sieghart, 1995; Dämgen and Lüddens, 1999).

### Implications for Z-Drugs Hypnotic Effect

While clinical studies observing the pharmacokinetics and pharmacodynamics of Z-drug mediated sleep are extensive, there have been relatively few studies comparing how hypnotic drugs target specific brain areas to induce sleep. Within the thalamus and hypothalamus are clusters of nuclei that relay information from subcortical structures to the cortex and both these regions are important for sleep-wake maintenance. The thalamic reticular nucleus generates characteristic sleep EEG firing rhythms, and the lateral hypothalamus is part of an ascending pathway stimulating cortical activity and wakefulness (Saper et al., 2001; Gent et al., 2018). Interestingly, eszopiclone but not zolpidem modulates GABAergic postsynaptic potentials in the thalamic reticular nucleus (Jia et al., 2009) and suppresses activity in the lateral hypothalamus (Kumar et al., 2011) to bring about sleep. Both of these regions contain a wider variety of GABA_*A*_ subunits including the γ3 subunit (Pirker et al., 2000) which may, in part, account for the differences. Compared to zolpidem, eszopiclone has a faster sleep onset, more time spent in the restorative non-rapid eye movement stage, and a differing EEG signature (Xi and Chase, 2008).

There is a need to understand how hypnotics mediate their effect to aid in future drug development. Z-drugs were designed well before our detailed understanding of GABA_*A*_ receptor subtypes (Bardone et al., 1978; Arbilla et al., 1985; Beer et al., 1997), and different GABA_*A*_ subunit preferences contribute to differences in drug action along with pharmacokinetic factors like plasma concentration and drug half-life. In this study, we limited receptors to only contain α1 in combination with γ1, γ2, or γ3. While Z-drugs preferentially modulate α1 receptors at low concentrations, at moderate to high concentrations they also modulate receptors with α2 and α3 subunits (Petroski et al., 2006; Nutt and Stahl, 2010; Ramerstorfer et al., 2010; Sieghart and Savic, 2018), and these subunits may also play a role in sleep generation (Kopp et al., 2004). In addition to α subunit preference variations, we provide evidence here that there are also differences in how Z-drugs modulate GABA_*A*_ receptors with γ3 subunits, but the significance of this *in vivo* is still unknown. In addition, future studies should characterize receptors with γ3 subunits in combination with α2/3.

The γ2 Phe77 mutation which abolishes zolpidem binding has been used as an *in vivo* pharmacogenetic model to explore zolpidem’s effects in particular brain regions (Wulff et al., 2007). This approach revealed that zolpidem specifically prolongs postsynaptic potentials within the hypothalamic tuberomammillary nucleus, reducing histamine levels across the brain sufficiently to induce sleep (Uygun et al., 2016). Because γ3-containing receptors are expressed within the same networks controlling sleep, elucidating any potential role they play would be important for the development of better hypnotics. Utilizing the approach of expressing the γ2 Phe77 mutation may reveal residual non-γ2 mediated behavioral effects related to zaleplon, indiplon, or eszopiclone administration. Moreover, because indiplon is efficacious on γ3, but not γ1-containing receptors, it would be well suited to specifically target γ3-containing receptors.

In conclusion, the approach taken of using concatenated GABA_*A*_ receptors has overcome issues of forming unexpected receptor populations when using single subunit cRNAs to express recombinant receptors in *X. laevis* oocytes. We used this strategy to clarify inconsistencies within the literature on what effects Z-drugs have on γ1- and γ3-containing GABA_*A*_ receptors. Using this strategy, we have shown that zaleplon, indiplon, and eszopiclone modulate γ3-containing GABA_*A*_ receptors with no effects on γ1-containing GABA_*A*_ receptors below 10 μM. Zolpidem and alpidem show no significant modulation on γ1 or γ3 subunits below 10 μM indicating that their pharmacological effects are likely limited to GABA_*A*_ receptors with γ2 subunits. Gaining a complete picture of the GABA_*A*_ receptor subtypes targeted by Z-drugs will help in the understanding of hypnotics and aid in developing drugs that more closely replicate physiological sleep with less adverse side effects.

## Data Availability Statement

The raw data supporting the conclusions of this article will be made available by the authors, without undue reservation.

## Ethics Statement

The animal study was reviewed and approved by The University of Sydney Animal Ethics Committee.

## Author Contributions

GR, VL, PA, and MC conceptualized the study and designed the experiments. GR, VL, and PA collected and analyzed the data. GR wrote the manuscript and prepared the figures. VL, PA, and MC reviewed the manuscript. All authors contributed to the article and approved the submitted version.

## Conflict of Interest

The authors declare that the research was conducted in the absence of any commercial or financial relationships that could be construed as a potential conflict of interest.
